# CRISPR-Cas9 screen in human embryonic stem cells to identify genes required for neural differentiation

**DOI:** 10.1016/j.xpro.2022.101682

**Published:** 2022-09-16

**Authors:** Shutao Qi, Sushama Sivakumar, Hongtao Yu

**Affiliations:** 1 Westlake Laboratory of Life Sciences and Biomedicine, Hangzhou, Zhejiang, China; 2 School of Life Sciences, Westlake University, Hangzhou, Zhejiang, China; 3 Department of Pharmacology, University of Texas Southwestern Medical Center, 6001 Forest Park Road, Dallas, TX 75390, USA

**Keywords:** Cell Biology, Developmental biology, CRISPR, Neuroscience, Stem Cells

## Abstract

Human embryonic stem cells (hESCs) continuously self-renew in culture and can be induced to differentiate into multiple cell types, including neural progenitor cells (NPCs). Here, we present a protocol to perform a CRISPR-Cas9 screen in hESCs to identify regulators that promote SOX1 expression during NPC formation. This screening protocol can be adapted with other endpoint reporters for the identification of genes involved in the commitment of hESCs to other cell lineages.

For complete details on the use and execution of this protocol, please refer to Sivakumar et al. (2022).

## Highlights

• Genome-wide CRISPR-Cas9 screen in human ESCs during neural differentiation
• Flow cytometry to isolate cells of interest for high-throughput genetic screens
• Antibody labeling of endogenous proteins bypasses the need for reporter cell lines
• The protocol is generally applicable to flow-cytometry-based CRISPR screens

## Abstract (editor highlights)

Publisher’s note: Undertaking any experimental protocol requires adherence to local institutional guidelines for laboratory safety and ethics.

## Graphical abstract

**Figure:**
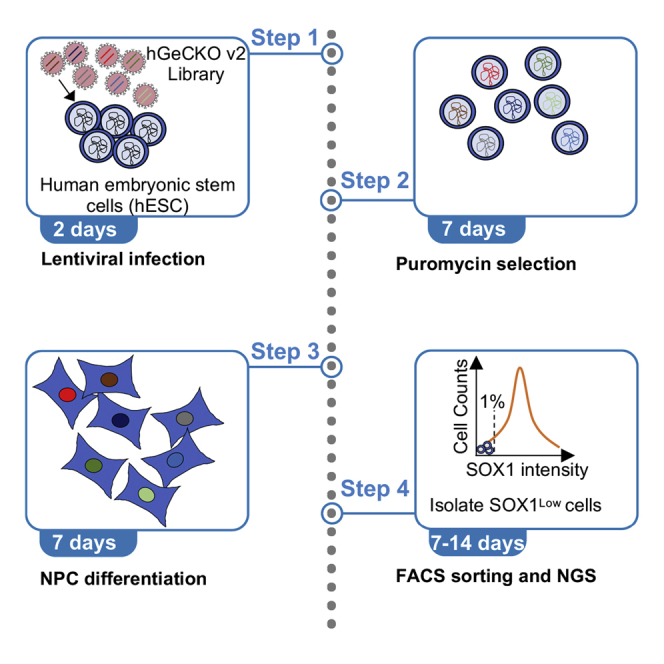

## Before you begin

**Figure 1:**
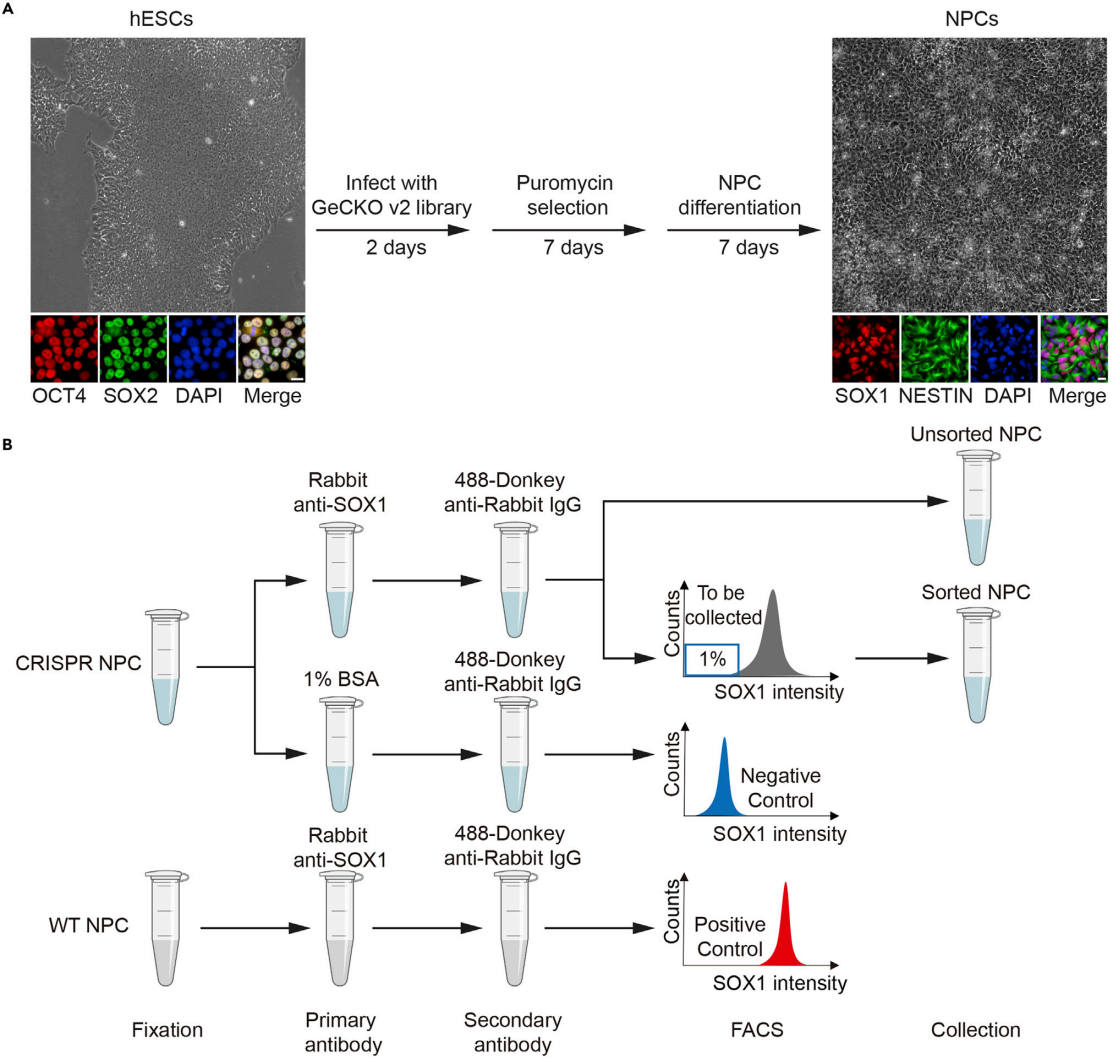
Workflow of the CRISPR-Cas9 screen for regulators of NPC differentiation (A) Wild type hESCs (OCT4^+^, SOX2^+^) were infected with the GeCKO v2 library and selected with puromycin for 7 days. After selection, the hESCs were differentiated into NPCs (SOX1^+^, NESTIN^+^) by culturing in the neural induction medium for another 7 days and subjected to FACS sorting. The SOX1^–^ cells were collected. Scale bar = 20 μm. (B) Experimental design of NPC staining and FACS sorting.

We adapted our CRISPR-Cas9 screening protocol from other published papers (Joung et al., 2017; Sanjana et al., 2014; Shalem et al., 2014). The overall screen strategy is outlined in Figure 1A. We used the GeCKO v2 knockout screening library that contained 6 unique sgRNAs to target all annotated genes in the human genome (Sanjana et al., 2014). Specifically, the library contained a total of 123,411 sgRNAs, which included sgRNAs targeting 19,050 genes, 2000 non-targeting control sgRNAs, and sgRNAs targeting 1,864 miRNAs. Using this library, we designed a CRISPR-Cas9 screen with an endpoint reporter to systemically identify genes required for NPC differentiation. Because CRISPR-Cas9 screens are expensive, labor intensive, and time consuming, it is critical to design effective screens with strong endpoints to ensure that the initial hits can be reproducibly validated.

### Institutional permissions

All human stem cell line work conducted in this protocol was under the oversight of the Stem Cell Research Oversight (SCRO) committee at University of Texas Southwestern Medical Center.

### GeCKO v2 sgRNA library amplification

**Timing: ∼2 days**

The original library obtained from Addgene (Catalog no: 1000000048) contains two sub-libraries (library A and B). Each sub-library contains 3 sgRNAs against each gene, with 65,383 sgRNAs in library A and 58,028 sgRNAs in library B. Each sub-library alone or both together can be used to perform screens. It is advantageous to use one sub-library if cells or reagents are limiting. Using both sub-libraries together will ensure maximum representation for each gene, and detection of multiple sgRNAs confirms the validity of each hit.

Each sub-library is provided as ∼1 μg plasmid with a concentration of 50 ng/μL. The first step is to amplify each plasmid sub-library separately without loss of representation of sgRNAs. Sufficient quantities of plasmid DNA are required to produce lentiviruses for the CRISPR-Cas9 screen. A high-quality plasmid library is essential to the success of the screen. Loss of representation of sgRNAs can occur during the transformation, bacteria culturing, or plasmid extraction steps. The plasmid amplification step should be performed carefully. Specifically, the GeCKO library needs to be electroporated into electrocompetent cells and cultured on solid culture plates. Chemical competent cells or liquid culture protocols should not be used. Their use may cause biased amplification and loss of representation of sgRNAs.

1. Prepare five Corning square bioassay dish LB agar plates (size: 245 mm × 245 mm) and three standard LB agar petri dish (size: 100 mm) containing 100 μg/mL ampicillin.

***Note:*** If the Corning square bioassay dishes are not available, each bioassay dish can be replaced with 10 standard petri dishes.

2. GeCKO v2 plasmid library transformation.
  a. Thaw the GeCKO v2 plasmid sub-libraries (A and B) on ice. Mix well by vortexing or pipetting. Spin briefly to collect all the contents to the bottom of the tube.
  b. For each sub-library, thaw one tube (100 μL) of Electromax Stbl4 competent cells on ice. Add 8 μL of plasmids (400 ng) to the competent cells. Mix gently with a P200 tip. Aliquot 25 μL/cuvette into four pre-chilled Bio-Rad electroporation cuvettes. Place the cuvettes on ice.
    ***Note:*** Other highly efficient electrocompetent cells, such as Endura™ Electrocompetent cells (Cat: #60242-1) from Lucigen, could be used as an alternative.
  c. Set the Bio-Rad electroporator program to Ec1 (1.8 kV). Take one cuvette each time and perform the electroporation procedure. Immediately add 1 mL pre-warmed SOC and transfer to a round-bottom 14 mL tube. Wash the cuvette one more time with 1 mL SOC and transfer to the 14 mL tube.
    ***Note:*** Electroporation is the preferred transformation method because of its high efficiency. Other transformation methods are not recommended as their low efficiency will lead to biased plasmid amplification and loss of representation of sgRNAs in the library.
  d. Repeat the electroporation procedure with the other three cuvettes. Pool the competent cells in 10 mL SOC.
  e. Distribute the 10 mL competent cells to two 14 mL tubes. Shake at 250 rpm for 1 h at 37°C. Meanwhile, prewarm five bioassay LB agar plates and three standard petri dishes containing ampicillin at 37°C.
3. Plate a dilution to calculate transformation efficiency.
  a. After the 1-h recovery period, pool the 10 mL transformed competent cells together and mix well.
  b. Remove 10 μL and add to 990 μL of LB medium. Mix well and remove 100 μL and add to 900 μL of LB medium. Plate 50 μL in one 10 cm petri dish to obtain 200,000-fold dilution of the transformed cells. In another 10 cm petri dish, plate 100 μL to obtain 100,000-fold dilution of the transformed cells. These petri dishes will be used to determine the transformation efficiency.
  c. Plate 100 μL of LB medium onto the third 10 cm petri dish plate as the negative control.
  d. Culture the petri dish plates at the same condition as the bioassay plates. Count the number of colonies and calculate the transformation efficiency to ensure that full library representation is preserved (see point 6 below).
4. Plate the transformations.
  a. Plate 2 mL of transformation on one bioassay plate and distribute evenly with a spreader until the liquid culture is largely absorbed into the agar.
  b. Plate all 10 mL of transformation on five Corning square bioassay plates.

***Note:*** It is important to spread transformations evenly to prevent unbalanced sgRNA distribution.

5. Culture all plates inverted for 12–14 h at 37°C.
6. Calculate transformation efficiency in dilution plates.
  a. Count the number of colonies on the dilution plates.
    ***Note:*** The colonies should be sparse. If bacteria are overgrown or the colonies are too dense, transformation must be repeated at a higher dilution to get individual colonies. This is essential to prevent biases in sgRNA distributions.
  b. Multiple the colony number by 100,000 or 200,000 for the total number of colonies of all transformations.
  c. Proceed if the total number of colonies exceeds 3 × 10^6^. This efficiency is equivalent to 50× colonies per construct in the GeCKO library.
    ***Note:*** Depending on the quality of electroporation or culturing, the number (N_dilution_) of colonies grown from each dilution petri dish could vary. The total number of colonies (N_total_) grown on the five bioassay plates can be calculated as: N_total_=N_dilution1_∗200,000 or N_dilution2_∗100,000. The coverage of each sgRNA in the library will be N_total_/123,411. If the number is greater than 50, the coverage should be sufficient for subsequent experiments.
7. Harvest colonies.
  a. Weigh 3–4 empty 50 mL Corning tubes and label the weight on each tube.
  b. Add 15 mL prechilled LB medium onto one bioassay plate. Scrape colonies with a spreader and transfer to a 50 mL tube on ice.
  c. Repeat scraping once with 15 mL fresh LB medium and combine all the cell suspension.
  d. Collect all the colonies from five plates and distribute the cell suspension to three 50 mL-tubes. Keep tubes on ice while doing this.
  e. Centrifuge the 50 mL tubes at 3,000 g for 10 min at 4°C. Discard the supernatant.
  f. Weigh the bacteria pellets in each tube and subtract the weight of the empty tube.

***Note:*** Typically, each tube contains 1–2 g bacterial pellets. Plasmid DNA can then be isolated using QIAGEN Maxiprep kit (use one column for each tube).

**Pause point:** Flash freeze pellets and store at −80°C or continue with plasmid extraction.

8. Plasmid extraction.
  a. Purify plasmids from bacteria pellets with the QIAGEN Maxiprep kit according to the manufacturer’s instructions, with two modifications:
    i. After adding chilled buffer P3 to lysate, centrifuge the conical tube to pellet lysed debris. Then, pour the clear supernatant into the barrel of the QIAfilter cartridge.
    ii. Warm the elution buffer to 50°C before the elution step to increase plasmid DNA yield during elution.
  b. Check the integrity of the library by running 1 μg of plasmids on 1% agarose gel.
    ***Note:*** Typically, a clear DNA band of >10 Kb can be seen on the gel without any recombinant smaller bands.
  c. Measure DNA concentration by a Nanodrop spectrophotometer.
    ***Note:*** Typically, 200–300 μg of plasmids can be extracted from 1 g of bacterial pellets.
  d. Store the library at −20°C.
    ***Note:*** The plasmid library can be stored at −20°C for at least 1 year.
    ***Note:*** High-throughput sequencing is recommended to confirm that most sgRNAs have good representation in the plasmid library.

### Lentivirus production

**Timing: ∼1 week**

The purpose of this step is to produce a complete GeCKO lentiviral library with full representation of 122,411 sgRNAs. HEK293FT cells are used to package lentiviral particles.

9. Maintenance of HEK293FT cells:
  a. Culture HEK293FT cells in complete DMEM medium containing 10% fetal bovine serum (FBS) and 1% PenStrep.
  b. Grow cells in a 37°C temperature-controlled incubator supplemented with 5% CO_2_.
  c. Passage cells when they reach ∼80% confluency.
10. To passage cells:
  a. Aspirate the spent medium and rinse cells by gently adding 5 mL of PBS.
  b. Remove the PBS and add 1 mL 0.05% Trypsin-EDTA.
  c. Incubate for 2–3 min at 37°C.
  d. Add 4 mL of warm DMEM complete medium to the dish and pipet gently to dissociate cells into single cell suspension.
  e. Transfer the cell suspension to a 15 mL Corning tube and centrifuge at 200 g for 5 min.
  f. Remove supernatant and resuspend cell pellets in 5 mL DMEM complete medium.
  g. Passage 1:10 into a new 10 cm dish.
11. Prepare cells for transfection:
  a. One day before transfection, seed 293FT cells into 10 cm dishes at 5 × 10^6^ cells/dish.
  b. Prepare 30 dishes for sgRNA library transfection to ensure optimal sgRNA distribution within the lentiviral library.
12. Lentivirus plasmid transfection. To produce lentiviruses, we transfected HEK293FT cells with both plasmid sub-libraries along with the envelope plasmid (pMD2.G) and the packaging plasmid (psPAX2).
  a. Prepare lentiviral plasmid mix for transfection as below:

**Table:** 

Component	1 dish	30 dishes
Opti-MEM	750 μL	22.5 mL
pMD2.G (envelope plasmid)	5 μg	150 μg
psPAX2 (packaging plasmid)	7.5 μg	225 μg
GeCKO-A sub-library	5 μg	150 μg
GeCKO-B sub-library	5 μg	150 μg
  b. Prepare the PLUS reagent mix as below:

**Table:** 

Component	1 dish	30 dishes
Opti-MEM	750 μL	22.5 mL
Plus Reagent	100 μL	3 mL
  c. Add the PLUS reagent mix to the lentivirus plasmid mix and incubate at room temperature (∼26°C) for 5 min.
  d. Prepare the Lipofectamine 2000 reagent as below:

**Table:** 

Component	1 dish	30 dishes
Opti-MEM	1.5 mL	45 mL
Lipofectamine 2000	50 μL	1.5 mL
  e. Add the lentiviral plasmids and the PLUS reagent mix to the Lipofectamine 2000 reagent, mix well, and incubate at room temperature (∼26°C) for 20 min.
  f. During the incubation, prepare HEK293FT cells for transfection. Replace growth media in 10 cm dishes with 8 mL Opti-MEM or serum free DMEM (without FBS).
  g. Add 3 mL of the transfection mixture to each HEK293FT dish.
  h. At 5–6 h after transfection, replace medium in each dish with 10 mL complete DMEM (containing FBS).
  i. Next day, add 340 μL 30% BSA to each dish to reach a final concentration of 1%.
    ***Note:*** Addition of 30% BSA is a low-cost equivalent to growing cells in high serum media and increases viral yield by at least 2-fold.
  j. At 48 h after transfection, harvest the first batch of viral supernatant.
  k. Add 7 mL of fresh DMEM complete medium supplemented with 1% BSA in each 10 cm dish and continue to culture cells in a 37°C CO_2_ incubator.
  l. At 72 h after transfection, harvest the second batch of viral supernatant.
13. Harvest, concentrate, and store lentiviruses.
  a. Pool the two batches of viral supernatant together. Aliquot into 50 mL Corning tubes.
  b. Centrifuge at 500 g for 10 min at room temperature. Transfer the viral supernatant to new 50 mL Corning tubes. Be careful to avoid cell debris at the bottom of the tubes.
  c. Add 1 volume of Lenti-X Concentrator to 3 volumes of viral supernatant. Mix well by gentle inversion. Incubate at 4°C for 12–16 h.
  d. Centrifuge virus/Lenti-X mix at 1,500 g for 50 min at 4°C. After centrifugation, a white pellet will be visible at the bottom of the tube.
  e. Carefully remove the supernatant, taking care not to disturb the pellet.
  f. Gently resuspend the pellet in 1/100th of the original supernatant volume using PBS.
  g. Pool all the concentrated viruses, mix well, and aliquot into small volumes. Store at −80°C. Avoid multiple freeze-thaw cycles.

### Coating 6-well plates with Matrigel

**Timing: 1 h**

Matrigel is a solubilized mixture of extracellular matrix proteins. It is essential to coat 6 well tissue culture plates in Matrigel to promote hESC maintenance, growth, and differentiation.

14. Thaw one tube of hESC-qualified Matrigel on ice for ∼1 h. Determine the dilution factor of Matrigel (listed on certificate of analysis) and use that volume to coat four 6-well tissue-culture-treated plates.
15. Place a 50 mL Corning tube on ice. Add 48 mL of cold DMEM/F12 medium. Using a prechilled pipette tip, transfer the appropriate volume of Matrigel to the cold DMEM/F12 medium. Pipette or invert to mix. Distribute 2 mL in each well of four 6-well plates.
16. Incubate at a 37°C incubator for at least 1 h before use.

***Note:*** The coated plates can be stored at 4°C or 37°C and should be used in one week. If stored at 4°C, the plates should be pre-warmed to room temperature (∼26°C) for at least 1 h before use.

### Determine the multiplicity of infection (MOI) of lentiviruses

**Timing: 1 week**

We will next measure the titer of the lentiviruses and determine the amount of viruses required to transduce the target cells. The goal is to ensure that each target cell is transduced with only one lentivirus copy (or one sgRNA/cell). If the concentration of lentiviruses during target cell infection is too high, more than one sgRNA will be delivered into every cell resulting in errors during computational analysis of Next Generation Sequencing (NGS) data. Hence it is critical to use a very low MOI (multiplicity of infection) during transduction. Generally, it is recommended to use a MOI ≤ 0.3 (transduction efficiency ≤ 30%) for *in vitro* cultured cell lines. However, for *in vivo* screens or if obtaining sufficient cell numbers is a challenge, MOI ≤ 1 (transduction efficiency ≤ 60%) is also acceptable.

We performed our CRISPR-Cas9 screen on human embryonic stem cells (hESCs). We used a female hESC line named WA09/H9. (Other hESC lines or hiPSC lines could also be used as alternatives. Their culturing and differentiation conditions should be optimized prior to screening.) hESCs continuously self-renew and are pluripotent, making them ideal to study human cell differentiation. We designed our CRISPR screen with hESCs to unravel new genes involved in neural development.

**Figure 2:**
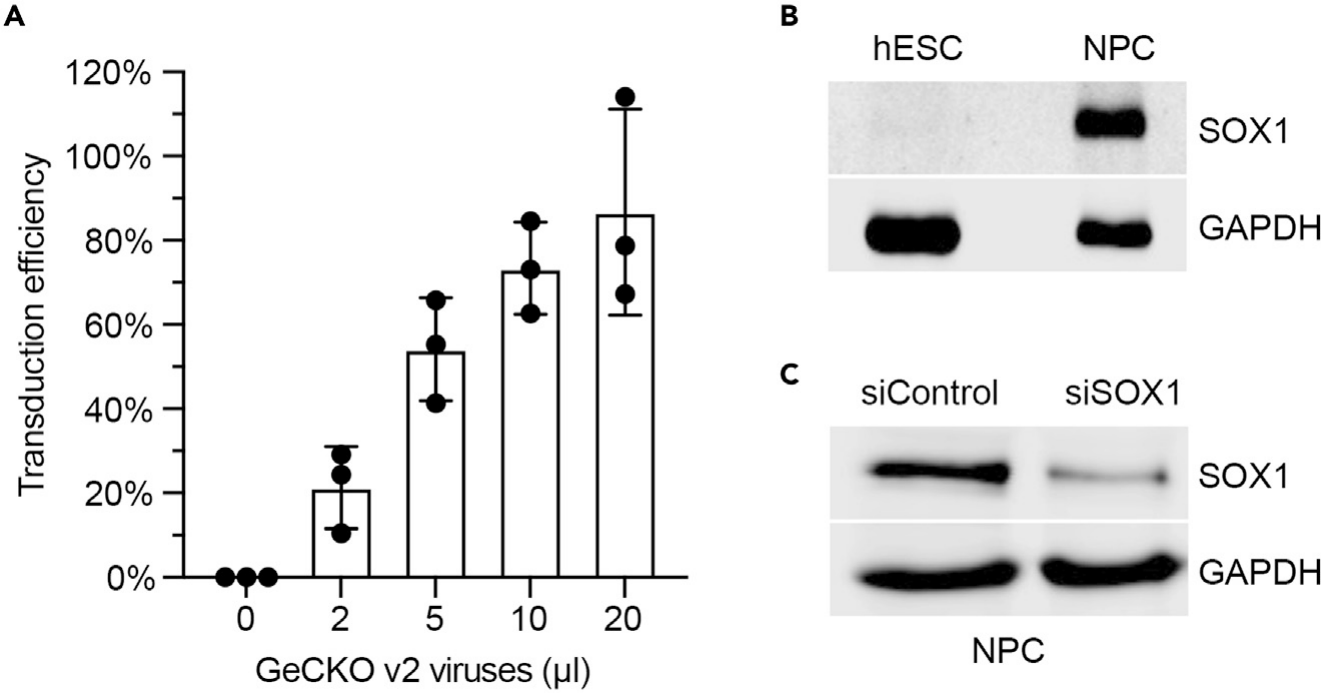
Optimization of the CRISPR screen (A) Measuring the transduction efficiency of different volumes of GeCKO v2 lentiviruses. Wild type hESCs were seeded in 6-well plates and transduced with different volumes of GeCKO v2 lentiviruses. At 48 h after transduction, cells were split into two wells and treated with or without puromycin. The transduction efficiency was calculated by comparing cell numbers in puromycin-treated or -untreated conditions. Mean ± SD; n = 3 independent experiments. (B) Western blot of whole cell lysates of hESCs or NPCs. (C) Western blot of whole cell lysates of NPCs transfected with the negative control siRNA or siRNA against the *SOX1* gene.
  h. Repeat the lentivirus transduction three times and determine the viral concentration required to obtain a transduction efficiency of ∼30%. This is considered as MOI = 0.3.

17. Culturing H9 hESCs.
  a. Remove a vial of H9 hESCs from liquid nitrogen storage.
  b. Thaw the vial in a water bath maintained at room temperature (∼26°C).
  c. When the vial is 90% thawed, resuspend freezing media in mTeSR1 media containing 10 μM ROCK inhibitor (Y-27632). Centrifuge cells at 300 g for 5 min.
  d. Aspirate media and resuspend cells in fresh mTeSR1 supplemented with 10 μM ROCK inhibitor (Y-27632). Pipette gently to maintain cells as clumps.
  e. Remove the DMEM/F12 medium from a Matrigel-coated plate. Be careful not to scratch the surface of the coated wells. Add H9 hESCs resuspended in mTeSR1 supplemented with ROCK inhibitor in 1–2 wells of the Matrigel-coated plate.
    ***Note:*** If the Matrigel-coated plates are stored at 4°C, pre-warm the plates to room temperature (∼26°C) for at least one hour before use.
  f. Incubate the plate in a 37°C incubator supplemented with 5% CO_2_.
  g. Refeed fresh mTeSR1 daily and passage cells when the well is ∼70% confluent.
  h. To passage cells, aspirate the medium and add 1 mL of pre-warmed Versene solution in each well. Incubate at 37°C for 8–10 min.
  i. Tap the plate gently to dissociate cells from the plate. Transfer cell suspension to a 15 mL Corning tube.
  j. Centrifuge the cell suspension at 200 g for 3 min. Aspirate the supernatant and resuspend the cell pellet in 1 mL mTeSR1 medium supplemented with 10 μM Y27632 ROCK inhibitor.
  k. Seed cells at 1:10 ratio into a new Matrigel-coated 6-well plate.
18. Prepare cells for transduction.
  a. Aspirate the spent medium. Rinse cells once with 1 mL/well pre-warmed DMEM/F12. Add 1 mL/well pre-warmed Accutase solution and incubate at 37°C for 8–10 min.
  b. Add 4 mL/well DMEM/F12. Pipette gently to dislodge cell aggregates. Transfer the cell suspension to a 15 mL Corning tube.
  c. Centrifuge the cell suspension at 200 g for 5 min.
  d. Remove the supernatant. Resuspend the cell pellet in 1 mL mTeSR1 supplemented with 10 μM Y27632 ROCK inhibitor. Count cell density with a cell counter.
  e. Add 1.5 mL/well mTeSR1 supplemented with 10 μM Y27632 to each well of a 6-well plate. Seed H9 ESCs at a density of 5 × 10^5^ cells/well in a 6-well plate.
  f. Add different amounts of viruses (1 μL, 2 μL, 5 μL, 10 μL, 20 μL, 50 μL, or 100 μL) to each well to determine the transduction efficiency. Mix by gently shaking the plate.
  g. Culture in a 37°C incubator supplemented with 5% CO_2_.
  h. At 24 h after transduction, refeed with fresh mTeSR1.

19. Calculate the transduction efficiency.
  a. At 48 h after transduction, dissociate cells with Accutase. Collect cell pellets and resuspend cells in mTeSR1.
  b. Count cell numbers and seed 4 × 10^5^ cells/well in duplicate wells of a new Matrigel-coated 6-well plate.
  c. Treat one well with 0.5 μg/mL puromycin. The other well serves as the control (without puromycin selection) to determine transduction efficiency.
    ***Note:*** The puromycin concentration needs to be optimized for different cell lines. For H9 ESCs, we have tested different puromycin concentrations and found that 0.5 μg/mL concentration works best. If using a different cell type other than H9 hESCs, the appropriate concentration of puromycin should be determined by performing a kill curve assay.
  d. At 24 h after puromycin treatment, refeed with fresh mTeSR1 supplemented with/without puromycin and Y27632.
  e. At 48 h after puromycin treatment, wash cells once with 1 mL/well DMEM/F12. Add 1 mL/well pre-warmed Accutase and incubate at 37°C for 10 min. Pipette gently to dislodge cell aggregates into single cell suspension.
  f. Count the cell number in each well using a cell counter. Repeat counting twice for each sample.
  g. For each viral concentration used, calculate the transduction efficiency by counting numbers of cells after puromycin selection divided by the number of cells in the wells without puromycin selection.
    ***Note:*** A linear relationship between viral volume and transduction efficiency is obtained at lower volumes, while saturation is achieved at higher viral volumes, as shown in Figure 2A.

## Key resources table

**Table:** 

REAGENT or RESOURCE	SOURCE	IDENTIFIER
**Antibodies**		

Rabbit polyclonal anti-SOX1 antibody	Cell Signaling Technology	Cat#4194S; RRID: AB_1904140
Rabbit monoclonal anti-GAPDH antibody	Cell Signaling Technology	Cat#2118; Clone 14C10; RRID: AB_561053
Alexa Fluor™ 488 Donkey Anti-rabbit IgG (H + L)	Thermo Fisher Scientific	Cat#A-21206; RRID: AB_2535792
Anti-rabbit IgG Dylight 800 Conjugate	Cell Signaling Technology	Cat#5151; RRID: AB_10697505

**Bacterial and virus strains**		

One Shot™ Stbl3™ Chemically Competent *E. coli*	Thermo Fisher Scientific	Cat#C737303
ElectroMAX™ Stbl4™ Competent Cells	Thermo Fisher Scientific	Cat#11635018

**Chemicals, peptides, and recombinant proteins**		

LB Broth Miller	Sigma-Aldrich	Cat#71753-6
LB Agar, Miller	Sigma-Aldrich	Cat#71752-6
SOC Outgrowth Medium	New England Biolab	Cat#B9020
Ampicillin sodium salt	Sigma-Aldrich	Cat#A9518-100G
DMEM	Thermo Fisher Scientific	Cat#11965-118
Opti-MEM™ I Reduced Serum Medium	Thermo Fisher Scientific	Cat#31985070
Fetal Bovine Serum	Sigma-Aldrich	Cat#F0926-500ML
L-Glutamine (200 mM)	Thermo Fisher Scientific	Cat# 25030081
PBS, pH7.4	Thermo Fisher Scientific	Cat#10010031
Penicillin-Streptomycin (10,000 U/mL)	Thermo Fisher Scientific	Cat#15140-163
0.05% Trypsin-EDTA	Thermo Fisher Scientific	Cat#25300-120
Lipofectamine 2000	Thermo Fisher Scientific	Cat#11668019
PLUS™ Reagent	Thermo Fisher Scientific	Cat#11514015
Lipofectamine RNAiMAX	Thermo Fisher Scientific	Cat#13778150
Bovine Serum Albumin solution	Sigma-Aldrich	Cat#A9576-50ML
BSA	Sigma-Aldrich	Cat#A7906-100G
Lenti-X Concentrator	Takara Bio	Cat#631232
Matrigel hESC-qualified matrix, LDEV free	Corning	Cat#354277
mTeSR1-cGMP	STEMCELL Technologies	Cat#85850
DMEM/F-12	Thermo Fisher Scientific	Cat#11320082
Versene solution	Thermo Fisher Scientific	Cat#15040066
Accutase	STEMCELL Technologies	Cat#07920
Y-27632 Rho/Rock pathway Inhibitor	STEMCELL Technologies	Cat#72308
Puromycin Dihydrochloride	Thermo Fisher Scientific	Cat#A1113803
STEMdiff™ SMADi Neural Induction Kit	STEMCELL Technologies	Cat#08581
BD Phosflow™ Fix Buffer I	BD Biosciences	Cat#557870
BD phosflow Perm buffer III	BD Biosciences	Cat#558050
Phenol solution	Sigma-Aldrich	Cat#P4557
UltraPure Phenol:Chloroform:Isoamyl Alcohol (25:24:1, v/v)	Thermo Fisher Scientific	Cat#15593031
Chloroform	Sigma-Aldrich	Cat#C2432
Glycoblue Coprecipitant	Thermo Fisher Scientific	Cat#AM9515
RNase A	QIAGEN	Cat#19101
Ethanol	Sigma-Aldrich	Cat#EX0276
Recombinant Proteinase K Solution (20 mg/mL)	Thermo Fisher Scientific	Cat#AM2548
Buffer AE	QIAGEN	Cat#19077
Herculase II Fusion DNA Polymerases	Agilent	Cat#600677
MgCl_2_ solution	New England Biolab	Cat#B0510AVIAL
DMSO	New England Biolab	Cat#B0515AVIAL
Agencourt AMPure XP Kit	Beckman Coulter	Cat#A63880

**Critical commercial assays**		

Qubit™ dsDNA BR Assay Kit	Thermo Fisher Scientific	Cat#Q32853
Qubit™ dsDNA HS and BR Assay Kits	Thermo Fisher Scientific	Cat#Q32854
HiSpeed Plasmid Maxi Kit	QIAGEN	Cat#12663
Agilent BioAnalyzer High Sensitivity DNA Analysis Kit	Agilent	Cat#5067-4626
KAPA Library Quantification Kit Illumina® Platforms	Roche	Cat#07960140001

**Experimental models: Cell lines**		

H9 human ES cell	WiCell	Cat#WA09
HEK293FT cell line	Thermo Fisher Scientific	Cat#R70007

**Oligonucleotides**		

Primer v2Adaptor-F: AATGGACTATC ATATGCTTACCGTAACTTGAAAGTATTTCG	This paper	N/A
Primer v2Adaptor-R: TCTACTATTCTTTCCC CTGCACTGTTGTGGGCGATGTGCGCTCTG	This paper	N/A
Primer-F01: AATGATACGGCGACCA CCGAGATCTACACTCTTTCCCTACA CGACGCTCTTCCGATCTTAAGTAGA GTCTTGTGGAAAGGACGAAACACCG	This paper	N/A
Primer-F02: AATGATACGGCGACCA CCGAGATCTACACTCTTTCCCTACA CGACGCTCTTCCGATCTATACACG ATCTCTTGTGGAAAGGACGAAACACCG	This paper	N/A
Primer-F03: AATGATACGGCGACCACC GAGATCTACACTCTTTCCCTACACGAC GCTCTTCCGATCTGATCGCGCGGTTC TTGTGGAAAGGACGAAACACCG	This paper	N/A
Primer-F04: AATGATACGGCGACCA CCGAGATCTACACTCTTTCCCTAC ACGACGCTCTTCCGATCTCGATCA TGATCGTCTTGTGGAAAGGACGAA ACACCG	This paper	N/A
Primer-F05: AATGATACGGC GACCACCGAGATCTACACT CTTTCCCTACACGACGCTCTTC CGATCTTCGATCGTTACCAT CTTGTGGAAAGGACGAAACACCG	This paper	N/A
Primer-F06: AATGATACGGCGACCACCGAG ATCTACACTCTTTCCCTACACGA CGCTCTTCCGATCTATCGATTCC TTGGTTCTTGTGGAAAGGACGA AACACCG	This paper	N/A
Primer-F07: AATGATAC GGCGACCACCGAGATCTA CACTCTTTCCCTACACGACGC TCTTCCGATCTGATCGATAAC GCATTTCTTGTGGAAAGGACG AAACACCG	This paper	N/A
Primer-F08: AATGATACG GCGACCACCGAGATCTAC ACTCTTTCCCTACACGACG CTCTTCCGATCTCGATCGA TACAGGTATTCTTGTGGAA AGGACGAAACACCG	This paper	N/A
Primer-F09: AATG ATACGGCGACCAC CGAGATCTACACTCT TTCCCTACACGACGC TCTTCCGATCTACGA TCGATAGGTAAGGTC TTGTGGAAAGGACGA AACACCG	This paper	N/A
Primer-F10: AATGATAC GGCGACCACCGAGATC TACACTCTTTCCCTACA CGACGCTCTTCCGATCT TAACAATGGTCTTGTGG AAAGGACGAAACACCG	This paper	N/A
Primer-F11: AATGATACG GCGACCACCGAGATCTA CACTCTTTCCCTACACGA CGCTCTTCCGATCTATAC TGTATCTCTTGTGGAAAG GACGAAACACCG	This paper	N/A
Primer-F12: AATGATACGG CGACCACCGAGATCTACA CTCTTTCCCTACACGACGC TCTTCCGATCTGATAGGTC GCATCTTGTGGAAAGGAC GAAACACCG	This paper	N/A
Primer-R01: CAAGCAGAAGA CGGCATACGAGATAAGTAGA GGTGACTGGAGTTCAGACGT GTGCTCTTCCGATCTtTCTACT ATTCTTTCCCCTGCACTGT	This paper	N/A
siControl: sense 5′-UCAUUCCGGAUA CUGCGAUUU dTdT-3′ antisense: 5′-AAAUCGCAGU AUCCGGAAUGA dTdT-3′	Sigma-Aldrich	N/A
siGENOME Human SOX1 siRNA SMARTpool	Dharmacon	Cat#M-012194-01-0005

**Recombinant DNA**		

LentiCRISPR v2	(Sanjana et al., 2014)	Addgene plasmid #52961
Human GeCKO v2 Library, 1-plasmid system	(Sanjana et al., 2014)	Addgene pooled library #1000000048
pMD2.G	Addgene	Addgene plasmid #12259
psPAX2	Addgene	Addgene plasmid #12260

**Software and algorithms**		

R Development Core	N/A	https://www.r-project.org
FastQC	N/A	http://www.bioinformatics.babraham.ac.uk/projects/fastqc
FastQ_Screen	N/A	http://www.bioinformatics.babraham.ac.uk/projects/fastq_screen
MAGeCK	(Li et al., 2014)	https://sourceforge.net/p/mageck/wiki/Home/
Prism 9	GraphPad	https://www.graphpad.com/scientific-software/prism/
Adobe Illustrator	Adobe	https://www.adobe.com/products/illustrator.html
Image studio Lite	LI-COR Biosciences	https://www.licor.com/bio/image-studio-lite/

**Other**		

Corning® square bioassay dishes	Corning	Cat#CLS431111
Gene Pulser Xcell system	Bio-Rad	N/A
TC20 automated cell counter	Bio-Rad	N/A
NanoDrop UV spectrophotometer	Thermo Fisher Scientific	N/A
Qubit 4 Fluorometer	Thermo Fisher Scientific	N/A
BD FACSAria™ Fusion Flow Cytometer	BD Biosciences	N/A
Thermal Mixer with Blocks	Thermo Fisher Scientific	N/A
Mastercycler® nexus - PCR Thermal Cycler	Eppendorf	N/A
NextSeq 500 high output sequencer	Illumina	N/A
Odyssey infrared imaging system	LI-COR Biosciences	N/A

## Materials and equipment

**Table:** DMEM complete medium

Reagent	Final concentration	Amount
DMEM	N/A	450 mL
FBS	10%	50 mL
L-Glutamine (200 mM)	2 mM	5 mL
Total	N/A	505 mL

***Note:*** The complete medium can be stored at 4°C for up to one month.

**Table:** mTeSR1 medium

Reagent	Final concentration	Amount
Basal medium	N/A	400 mL
5× Supplement	1×	100 mL
Penicillin-Streptomycin (10,000 U/mL)	100 U	5 mL
Total	N/A	505 mL

***Note:*** The complete medium can be stored at 4°C for up to two weeks. If not used immediately, the medium can be aliquoted to small volumes and stored at −20°C for up to 6 months.

**Table:** SMADi neural induction medium

Reagent	Final concentration	Amount
Neural induction basal medium	N/A	250 mL
Dual SMADi Supplement (500×)	1×	500 μL
Penicillin-Streptomycin (10,000 U/mL)	100 U	2.5 mL
Total	N/A	253 mL

***Note:*** The complete medium can be stored at 4°C for up to two weeks. If not used immediately, the medium can be aliquoted to small volumes and stored at −20°C for up to 6 months.

**Table:** STE lysis buffer

Reagent	Final concentration	Amount
0.5 M EDTA (pH8.0)	10 mM	200 μL
1 M Tris-HCl (pH8.0)	10 mM	100 μL
5 M NaCl	100 mM	200 μL
Proteinase K (20 mg/mL)	200 μg/mL	100 μL
10% SDS	0.4%	400 μL
Nuclease free water	N/A	9 mL
Total	N/A	10 mL

***Note:*** Prepare the buffer fresh before use.

## Step-by-step method details

### GeCKO v2 lentiviral transduction for screen

**Timing: 2–3 weeks**

The lentiviral GeCKO v2 library will now be used to transduce H9 ESCs. Considering the low transduction efficiency (∼30% if MOI = 0.3), at least 120 million cells are required to maintain enough coverage of the library. After transduction, the cells should be treated with puromycin for at least one week to kill non-transduced cells and promote gene editing.

1. Prepare H9 ESCs for transduction.
  a. Culture H9 ESCs in ten 6-well plates. Refeed fresh mTeSR1 daily until cells reach ∼70% confluency.
  b. Remove the spent medium. Rinse cells with 1 mL/well DMEM/F12 and add 1 mL/well Accutase solution. Incubate at 37°C for 10 min.
  c. Add 4 mL/well DMEM/F12. Pipette gently to dislodge cell aggregate into single cell suspension. Pool cell suspension from one plate to a 50 mL Corning tube.
  d. Centrifuge the cell suspension at 200 g for 5 min.
  e. Remove the supernatant. Resuspend cell pellets in 10 mL/tube mTeSR1 supplemented with 10 μM Y27632 ROCK inhibitor.
    i. Pool all cell suspension together and mix well by pipetting.
    ii. Count cell numbers with a cell counter.
2. Transduction of cells.
  a. Prepare the transduction master mix by combining 120 million cells with the appropriate volume of viruses to achieve the desired MOI of 0.3.
  b. Seed cells at 5 × 10^5^ cells/well in a 6-well plate. Use 40 Matrigel-coated 6-well plates to seed all 120 million cells.
  c. Gently shake 6-well plates back and forth to spread cells evenly.
  d. Culture cells in a 37°C incubator.
3. Puromycin selection and maintenance of transduced cells.
  a. Refeed cells daily with 1.5 mL/well fresh mTeSR1 supplemented with 0.5 μg/mL puromycin.
  b. Keep puromycin treatment for 7 days. Puromycin will kill uninfected H9 ESCs and promote growth of H9 ESCs infected with the lentiviral GeCKO library.
  c. Passage cells when they are 70% confluent.
    i. When passaging, dissociate cells with Accutase, pool all cells, re-suspend in mTeSR1 supplemented with 10 μM Y27632 ROCK inhibitor and 0.5 μg/mL puromycin, mix thoroughly and count cell density.
    ii. Seed cells at 3 × 10^5^ cells/well into new Matrigel-coated 6-well plates. Seed at least 36 million cells every time to maintain 300× coverage. This ensures optimal sgRNA representation in cells.

### NPC induction and harvesting

**Timing: 1–2 weeks**

In the Sivakumar et al. (2022) study, we performed a genetic screen to unravel novel regulators of neural differentiation. We used SOX1, a well-established neural progenitor cell (NPC) marker, as an end point reporter in our genetic screen. The SOX1 protein is not expressed in ESCs but is robustly expressed in NPCs, making it an ideal marker for NPC development (Figure 2B). SOX1-positive NPCs were also functional and could be efficiently differentiated into neurons or astrocytes. We differentiated H9 ESCs infected with the GeCKO lentiviral library into NPCs by culturing them in SMADi neural induction medium. After 7 days of differentiation, NPCs were harvested and fixed for SOX1 staining with a specific SOX1 antibody (Figure 2C). Other NPC markers, such as PAX6 or NESTIN, could also be used if the antibodies are specific.

4. Wash H9 ESCs (the uninfected control cells and cells infected with the GeCKO lentiviral library) once with 1 mL/well warm DMEM/F12. Add 1 mL/well warm Accutase. Incubate at 37°C for 10 min.
5. Add 4 mL/well warm DMEM/F12. Pipette gently to dislodge cell aggregates into single cells. Transfer the cell suspension to 50 mL tubes.
6. Centrifuge at 200 g for 5 min.
7. Remove the supernatant. Resuspend cell pellets in 10 mL SMADi Neural induction medium supplemented with 10 μM Y27632 ROCK inhibitor. Pool all infected cells, mix thoroughly, and count cell density in the suspension.
8. Seed cells at 2 × 10^6^ cells/well in five Matrigel-coated 6-well plates. Culture in a 37°C incubator.
9. From day 1 to day 7 of NPC differentiation, refeed cells every day with 2 mL/well fresh SMADi Neural induction medium.
10. On day 7 of NPC induction, cells are ready for harvesting.
  a. To harvest cells, rinse cells with warm DMEM/F12 and dissociate cells by incubating with 1 mL/well Accutase for 10 min at 37°C.
  b. Pool all cells of the same condition together and count the cell density.
  c. Centrifuge cells at 300 g for 5 min.
  d. Resuspend cell pellets in 20–30 mL cold Cytofix I buffer to fix cells for FACS sorting and staining. Incubate at room temperature (∼26°C) for 20 min.

***Alternatives:*** Other commercial or homemade fixation buffers could also be used.

11. Centrifuge at 1,000 g for 10 min. Remove the Cytofix buffer and wash with 40 mL of cold PBS.
12. Centrifuge at 1,000 g for 10 min. Remove PBS and re-suspend cell pellets in 20–30 mL cold Perm-III buffer.

***Note:*** The Perm-III buffer promotes permeabilization of cell membranes for antibodies to enter cells during staining protocols.

***Alternatives:*** Other commercial or homemade permeabilization buffers could also be used.

13. Incubate cells for 30 min on ice.

***Note:*** Alternatively, cells can be stored in the Perm-III buffer for up to 6 months at −20°C.

### NPC staining and FACS sorting

**Timing: 2 days**

We stained day 7 differentiated cells (both the uninfected control cells and cells infected with the library) with the NPC specific marker SOX1 and used FACS to sort cells based on the SOX1 signal. The uninfected cells served as a positive control and exhibited SOX1-positive staining (Figure 1B).

14. Centrifuge NPC cells in the Perm-III buffer for 10 min at 1,000 g.
15. Remove the supernatant and resuspend cell pellets in 30 mL cold PBS containing1% BSA. Centrifuge at 1,000 g for 10 min to collect cell pellets. Repeat this twice.
16. Re-suspend cell pellets in 7 mL PBS containing 1% BSA. Save 50 μL cell suspension as unstained control. Save 50 μL of cell suspension as secondary-antibody control.
17. Add 50 μL SOX1 antibody to cell suspension (∼1:150 dilution).
18. Incubate at room temperature (∼26°C) with rotation for 1 h.
19. Wash cells twice with PBS containing 1% BSA.
20. Resuspend cell pellets in 7 mL PBS containing 1% BSA and add 15 μL 488-conjugated secondary antibody (∼1:500 dilution).
21. To 50 μL secondary-antibody control cells, add 450 μL PBS containing 1% BSA and 1 μL 488-conjugated secondary antibody.
22. Incubate cells with the secondary antibody at room temperature (∼26°C) with rotation for 1 h.
23. Wash cells twice with PBS containing 1% BSA.
24. Filter cell suspension with 70-μm cell strainers to remove any debris before running samples on a FACS sorter.
25. Count filtered cells and set aside 3.6 × 10^7^ cells as the unsorted control.

***Note:*** The unsorted control is essential for identifying screen hits during NGS analysis.

26. Subject other cells to FACS sorting.
  a. Use the unstained control sample and the secondary-antibody control sample as SOX1-negative populations. Use the uninfected stem cells differentiated into day 7 NPCs as positive controls for SOX1 expression. The positive and negative controls were used to gate the NPC population in cells infected with the GeCKO library.
  b. Harvest the bottom 1% of NPCs with the lowest SOX1 signal.
27. Collect cell pellets of the sorted SOX1-negative NPCs.
28. Flash freeze pellets in liquid nitrogen and store them at −80°C.

### Isolate genomic DNA from sorted or unsorted NPCs

**Timing: 2 days**

The next step is to extract the genomic DNA from sorted or unsorted NPCs.

29. Thaw cell pellets on ice. Resuspend in the STE lysis buffer (1 mL per 1 × 10^7^ cells).
30. Aliquot 500 μL cell suspension into each microtube and incubate 12–16 h at 56°C in a thermal mixer.
31. After incubation, cool down the cell lysate to room temperature. Add 4 μL (400 μg) RNase A and incubate for 1 h at 37°C in a thermal mixer.
32. Add 500 μL buffer-saturated phenol (pH 7.9) to each tube and mix by vortexing for 1 min.
33. Centrifuge at 13,000 rpm for 5 min. Transfer the aqueous phase to new tubes (∼550 μL).
34. Add equal volume of phenol:chloroform:isoamyl alcohol and mix by vortexing.
35. Centrifuge at 13,000 rpm for 5 min and transfer the aqueous phase to new tubes (∼550 μL).
36. Add equal volume of chloroform and mix by vortexing.
37. Centrifuge at 13,000 rpm for 5 min. Transfer the aqueous phase (∼300 μL) to new tubes. Add 0.5 μL Glycoblue.

***Note:*** Glycoblue labels genomic DNA blue and helps visualize the genomic DNA pellet when the yield is very low. It is an optional step and can be skipped if the yield is high.

38. Add 900 μL ethanol (the final centration is 75%) and mix by inverting.
39. Freeze at −80°C for 1 h to 12 h.
40. Centrifuge at 4°C for 10 min and discard the supernatant.
41. Wash once with 1 mL 75% ethanol.
42. Air dry for 10–15 min at room temperature.
43. Add 50 μL AE buffer (10 mM Tris-Cl, 5 mM EDTA; pH 9.0) to each tube.
44. Incubate at 37°C for 5 h up to 12 h to dissolve DNA.

***Alternatives:*** Genomic DNA can also be eluted with nuclease-free water.

45. Measure the DNA concentration with a Nanodrop spectrophotometer.
46. Store DNA at −20°C.

### Amplify the sgRNA cassette from genomic DNA by PCR

**Timing: 1 week**

The purpose of this step is to amplify the sgRNA-containing cassette and add NGS adaptors for high-throughput sequencing.

47. Perform the first round PCR to amplify the sgRNA-containing cassette from genomic DNA of unsorted and sorted NPCs:
  a. Set up each PCR reaction as follows:

**Table:** 

Components	Amount per reaction
5× Herculase II Reaction Buffer	10 μL
dNTP mix (25 mM each dNTP)	0.5 μL
V2 adaptor-F (10 μM)	2.5 μL
V2 adaptor-R (10 μM)	2.5 μL
MgCl_2_ (50 mM)	1.5 μL
DMSO	1.5 μL
Genomic DNA	2 μg
Herculase II Fusion DNA Polymerase	0.5 μL
H_2_O	To 50 μL

    **CRITICAL:** It is important to use enough genomic DNA for PCR to maintain the coverage of the sgRNA library. For unsorted NPCs, the yield of gDNA from 3.6 × 10^7^ cells is at least 200 μg. Set up 50 μL PCR reaction for every 2 μg gDNA. Hence, for 200 μg gDNA, 100 PCR reactions were set up. For the sorted NPCs, we divided all the gDNA samples into 3 PCR reactions.
    ***Alternatives:*** Other high fidelity DNA polymerases can also be used for PCR.
  b. Perform PCR using the following conditions:

**Table:** 

Steps	Temperature	Time	Cycles
Initial denaturation	98°C	30 s	1
Denaturation	98°C	1 s	20 cycles
Annealing	55°C	5 s	
Extension	72°C	35 s	
Final extension	72°C	1 min	1
Hold	10°C	Forever
  c. After the PCR reaction, pool all PCR products from the same genomic DNA sample. Mix thoroughly and either store at −20°C or use as template for the second round of PCR.
    **Pause point:** The PCR product can be stored at −20°C if not performing the second round PCR immediately.
48. Perform a second round of PCR to add NGS adaptors.
  a. Set up PCR reaction as follows:

**Table:** 

Components	Amount per reaction
5× Herculase II Reaction Buffer	10 μL
dNTP mix (25 mM each dNTP)	0.5 μL
F01-F12 (10 μM)	2.5 μL
R01 (10 μM)	2.5 μL
MgCl_2_ (50 mM)	1.5 μL
DMSO	1.5 μL
First round PCR product	2.5 μL
Herculase II Fusion DNA Polymerase	0.5 μL
H_2_O	28.5 μL

    **CRITICAL:** To maintain the coverage of sgRNA library, perform one PCR reaction for every 5,000 sgRNAs. For the unsorted NPCs, we performed 30 PCR reactions (24 reactions is enough). For the sorted NPCs, we performed 6 PCR reactions.
    ***Note:*** Each forward primer (F01 to F12) contains an 8-bp barcode as well as staggered sequences with different sequence and length. The barcodes are used to discriminate different samples when doing multiplex sequencing. The staggered sequence is used to introduce length diversity to the sequencing library. It is recommended to pool multiple forward primers with different barcodes and staggered sequences and use the primer mix for the PCR reaction.
  b. Perform PCR using the following conditions:

**Table:** 

Steps	Temperature	Time	Cycles
Initial denaturation	98°C	30 s	1
Denaturation	98°C	1 s	10–14 cycles
Annealing	55°C	5 s	
Extension	72°C	35 s	
Final extension	72°C	1 min	1
Hold	10°C	Forever

    **CRITICAL:** The number of PCR cycles for each sample should be optimized to produce enough target DNA for sequencing while minimizing PCR amplification biases. Usually, 10–14 cycles will be sufficient.
  c. Pool PCR products from the same DNA template. Run a 10 μL sample on 1% agarose gel to check the target DNA band.
    ***Note:*** After the second round of PCR, one specific DNA band around 300 bp should be observed.
    **Pause point:** The PCR product can be stored at −20°C if it is not purified immediately.

49. PCR product purification with AMPure XP beads.
  a. Mix the AMPure beads thoroughly by inverting.
  b. Combine 100 μL PCR product with 180 μL AMPure beads in a PCR tube. Mix by pipetting gently.
  c. Place the PCR tube on a magnetic rack and incubate for 2 min at room temperature.
  d. Aspirate the supernatant carefully without disturbing the beads. Wash beads with 200 μL 70% ethanol and mix by pipetting gently. Place the tube on the magnetic rack for 2 min.
  e. Aspirate the supernatant carefully without disturbing the beads. Repeat 70% ethanol washing two more times. Place the tube on the magnetic rack for 2 min.
  f. Aspirate the supernatant carefully without disturbing the beads. Air dry beads for 8 min. Add 40 μL nuclease-free water and incubate for 3 min at room temperature.
  g. Place the tube on the magnetic rack, incubate for 2 min, and transfer the supernatant to a new 1.5 mL tube.
  h. Store the purified DNA at −20°C.

### High-throughput sequencing and data analysis

**Timing: 3–4 weeks**

The purpose of this step is to use high throughput sequencing to analyze all sgRNAs that are integrated in the genome. By comparing the distribution of sgRNAs in the unsorted and sorted NPCs, we can determine whether a given sgRNA is enriched or depleted in the target cell population.

50. Check the size of DNA fragments with the Agilent Bioanalyzer High Sensitivity DNA Analysis Kit.
51. Measure DNA concentration both by the Qubit dsDNA HS assay kit (Thermo Fisher Scientific) and by qPCR using the KAPA Library Quantification Kit (Roche) for Illumina platforms.
52. Pool all samples and sequence on Illumina NextSeq 500 sequencer or other equivalent instrument with read configuration as 75-bp single-end sequencing. Sequence 20–30 million reads per sample.
53. Analyze the sequencing data with the MAGeCK algorithm (Li et al., 2014).

***Note:*** The reference sgRNA library sequences for human GeCKO v2.0 (A and B) were downloaded from Addgene (https://www.addgene.org/pooled-library/). The clean sequence reads were mapped to the reference sgRNA library with mismatch option as 0 using MAGeCK. Read counts for each sgRNA were generated and median normalization was performed to adjust for library size. Positively and negatively selected sgRNAs and genes were identified with MAGeCK using the default parameters.

## Expected outcomes

An example of expected outcomes of an NPC differentiation screen can be found in the original article by Sivakumar et al. (2022). The raw and analyzed sequencing data can be downloaded from GEO: GSE168587. The reference sgRNA library file can be downloaded from Addgene (https://www.addgene.org/pooled-library/).

## Limitations

With the current protocol by using the SOX1 signal as a readout, positive regulators that promote NPC induction from ESCs are successfully unraveled. This FACS-based screen strategy can also be applied to study novel regulators that control the differentiation of other cell types. This FACS-based screen relies on the specificity of the antibody. One needs to test the antibody specificity before the screen. It is also imperative to use a reporter that is a well-established marker for a particular cell type. After much deliberation, we decided to use SOX1 as a reporter for our screen, because SOX1 is not expressed in stem cells and is highly expressed in NPCs. In certain cases, it might not be enough to use one single marker gene to indicate the target cell type. One may identify regulators that promote the expression of the marker gene, but not the differentiation of the target cell type. In this case, the screen should be performed using multiple markers as readout.

## Troubleshooting

### Problem 1

When running plasmids library on 1% agarose gel, a smaller band of ∼1 kb can be seen in addition to the expected >10 kb band (before you begin step 8).

### Potential solution

The smaller band is caused by plasmid recombination between the lentiviral long-terminal repeats. To reduce plasmid recombination, the bacteria can be cultured at 32°C instead of 37°C.

### Problem 2

Severe cell death after lentiviral transduction (before you begin step 19).

### Potential solution

Too high a titer of lentiviruses may cause severe cell death even without puromycin treatment. It is important to transduce cells with low transduction efficiency (transduction efficiency ∼30%).

### Problem 3

Too much cell death when performing Puromycin selection (before you begin step 19).

### Potential solution

The sensitivity to puromycin varies greatly for different cell types. It is essential to generate a kill curve to determine the optimal puromycin concentration for the target ES cells. Moreover, the transduced ES cells tend to undergo apoptosis when the surrounded non-transduced cells are killed by puromycin. It is recommended to treat cells with the Y27632 ROCK inhibitor during the first two days of puromycin selection.

### Problem 4

Too much spontaneous differentiation during hESC culturing (before you begin step 17).

### Potential solution

hESCs need to be maintained carefully and refed daily. High cell confluency or insufficient nutrient supply may cause hESCs to differentiate spontaneously. It is critical to refeed hESCs with sufficient medium and passage cells when they grow to ∼70% confluency.

### Problem 5

Non-specific bands during PCR amplification of the sgRNA library from genomic DNA (major step 49).

### Potential solution

Non-specific bands can be observed with too many PCR cycles. One can optimize the PCR specificity by reducing the PCR cycles. If the non-specific bands persist, gel extraction could be used to purify only the desired PCR product.

## Resource availability

### Lead contact

Further information and requests for resources and reagents should be directed to and will be fulfilled by the lead contact, Hongtao Yu (yuhongtao@westlake.edu.cn).

### Materials availability

Plasmids and cell lines described in this study will be made available upon request.

## Data Availability

The data generated during this study are available at GEO: GSE168587.
